# Combination Strategies to Optimize Efficacy of Dendritic Cell-Based Immunotherapy

**DOI:** 10.3389/fimmu.2018.02759

**Published:** 2018-12-05

**Authors:** Mandy van Gulijk, Floris Dammeijer, Joachim G. J. V. Aerts, Heleen Vroman

**Affiliations:** ^1^Department of Pulmonary Medicine, Erasmus MC, Rotterdam, Netherlands; ^2^Erasmus Cancer Institute, Erasmus MC, Rotterdam, Netherlands

**Keywords:** DC-therapy, combination therapy, chemotherapy, radiotherapy, immune checkpoint inhibitors

## Abstract

Dendritic cells (DCs) are antigen-presenting cells (APCs) that are essential for the activation of immune responses. In various malignancies, these immunostimulatory properties are exploited by DC-therapy, aiming at the induction of effective anti-tumor immunity by vaccination with *ex vivo* antigen-loaded DCs. Depending on the type of DC-therapy used, long-term clinical efficacy upon DC-therapy remains restricted to a proportion of patients, likely due to lack of immunogenicity of tumor cells, presence of a stromal compartment, and the suppressive tumor microenvironment (TME), thereby leading to the development of resistance. In order to circumvent tumor-induced suppressive mechanisms and unleash the full potential of DC-therapy, considerable efforts have been made to combine DC-therapy with chemotherapy, radiotherapy or with checkpoint inhibitors. These combination strategies could enhance tumor immunogenicity, stimulate endogenous DCs following immunogenic cell death, improve infiltration of cytotoxic T lymphocytes (CTLs) or specifically deplete immunosuppressive cells in the TME, such as regulatory T-cells and myeloid-derived suppressor cells. In this review, different strategies of combining DC-therapy with immunomodulatory treatments will be discussed. These strategies and insights will improve and guide DC-based combination immunotherapies with the aim of further improving patient prognosis and care.

## Introduction

Dendritic cells (DCs) are the most potent antigen-presenting cells (APCs) and mediate a critical role in the interface between the innate and adaptive immune system. DCs can be subdivided in different subsets including conventional DCs (cDCs) and plasmacytoid DCs (pDCs) that arise in the bone marrow and reside in peripheral tissues in an immature state. In addition, monocytes are able to differentiate into monocyte-derived DCs (moDCs) upon inflammatory conditions (1–4). Activation and maturation of DCs are induced upon exposure to environmental stimuli including damage-associated molecular patterns (DAMPs) and pathogen-associated molecular patterns (PAMPs), leading to enhanced expression of co-stimulatory molecules, cytokine production, reduced phagocytosing capacity, and improved T- and B-cell activation (5, 6). DC-mediated T-cell activation is initiated by antigen presentation on major histocompatibility class (MHC) I and II and further guided by co-stimulation and secretion of cytokines (7–9). In addition to T-cell activation, DCs can activate natural killer (NK) cells by cell-cell contacts and secretion of pro-inflammatory cytokines such as type I interferons (IFNs) (10). However, in a tumor setting, DC functionality is often compromised as, for example, oncogenic mutations limit DC migration (11–14). In addition, factors secreted by cancer cells limit DC maturation by inducing overexpression of signal transducer and activation of transcription 3 (STAT-3) (15). This leads to insufficient antigen presentation, T-cell anergy and decreased T-cell proliferation, thereby restricting effective anti-tumor immunity (16–18).

Therefore, administering mature *ex vivo*-activated DCs loaded with tumor antigens may circumvent suppressive tumor-derived signals, thereby inducing effective anti-tumor immunity upon vaccination. For the past two decades, DC-therapy has shown to be safe, well-tolerated and capable of inducing anti-tumor immunity (19). However, response rates to DC-therapy are limited, with objective responses rarely exceeding 15% (20). Several mechanisms may contribute to the limited clinical efficacy besides suboptimal DC-therapy design, including downregulation of tumor-associated antigens (TAAs) and MHC molecules by tumor cells, restricted migration of DCs to lymph nodes (LN) and the inherent immune suppressive tumor microenvironment (TME) (21–26). The TME harbors a complex network of tumor tissue, stroma and immune cells including tumor-associated macrophages (TAMs), myeloid-derived suppressor cells (MDSCs), and regulatory T-cells (Tregs). These suppressive cells inhibit activation, proliferation and effector functions of infiltrating immune cells by the expression of co-inhibitory molecules and secretion of immunosuppressive cytokines (27–29). Conventional therapies, including chemotherapy and radiotherapy, or more recently developed immunotherapies such as immune checkpoint inhibitors are able to counteract the immunosuppressive environment of the tumor. Therefore, combining these therapies with DC-therapy could lead to synergistic effects and improve clinical responses. In this review, we will discuss current approaches of DC-therapy, promising combinations with chemotherapy, radiotherapy, and immune checkpoint inhibitors that are clinically applicable and future perspectives for novel combination therapies that can improve DC-therapy efficacy.

## Current Approaches of DC-Therapy

In order to obtain a sufficient number of DCs for administration, DCs are commonly generated from isolated CD14^+^ monocytes or from CD34^+^ hematopoietic progenitors isolated from peripheral blood, bone marrow or cord blood (3, 5). Culturing purified CD14^+^ monocytes with granulocyte-monocyte derived growth factor (GM-CSF) and interleukin (IL) 4 will lead to differentiation into immature moDCs (30). Vaccination with these immature DCs loaded with tumor antigens characterizes first-generation DC-therapy and resulted in poor clinical results with a tumor regression of 3.3% (31). In second-generation DC-therapy, DCs are additionally matured by ‘maturation cocktails’ including Toll-like receptor ligands and cytokines which improved clinical results with objective response rates of 8–15% (31). Sipuleucel-T, the only US FDA approved DC-therapy for use in (prostate) cancer patients, can be positioned at the intersection between first- and second-generation DC-therapy as maturation is not achieved by maturation cocktails but rather by the fusion of GM-CSF to prostate antigen (32). In next generation DC-therapy, naturally-occurring DC (nDCs) subsets are employed as nDCs are superior over moDCs in terms of functionality and production costs and time. In addition, different DC subsets also induce different tumor-specific immune responses, as vaccination with murine cDC1s induced a prominent CD8^+^ T-cell driven anti-tumor immune response that was beneficial in tumors with abundant Tregs whereas cDC2s induced a Th17-mediated anti-tumor immune response that was advantageous in tumors with TAMs (33, 34). Clinical trials using nDCs have shown that the usage of nDCs is safe, feasible and associated with promising efficacy, which indicates that this should be further investigated (35, 36).

### DC Loading

DCs can be loaded with different sources of tumor antigens, such as mRNA, peptides, proteins or whole tumor cell lysate (5, 37). While peptides bind directly to MHC molecules, proteins and tumor cells must be phagocytosed and processed before presentation on MHC molecules can occur. Furthermore, loading of DCs with tumor-associated peptides enables the induction of specific T-cell responses, thereby minimizing the risk on side-effects. However, for most tumor types, TAAs are still unidentified. Loading the DCs with tumor lysate circumvents the requirement of identified TAAs and additionally initiates a broad spectrum of immune responses that is not restricted to cytotoxic T lymphocyte (CTL) activation. This can improve DC-therapy efficacy as objective clinical responses observed upon treatment with DCs loaded with tumor lysate (8.3%) are higher than treatment with DCs presenting defined antigens (3.6%) in a meta-analysis of 173 trials (38).

### Route of Administration

To induce effective anti-tumor immunity, migration of DCs to lymph nodes is essential. Therefore, various administration routes have been exploited (intradermally, intranodally, intravenously, subcutaneously, and intratumorally), although to date the superior route of administration is still not established. Also the percentages of DCs that migrate successfully toward the lymph nodes is limited, with up to 4% of injected DCs reaching the lymph node after intradermal injection and 0–56% reaching the lymph node after intranodal injection (26). The migratory capacity can be improved by preconditioning the injection site with a potent recall antigen, tetanus/dipteria toxoid, which improved overall survival (OS) and progression free survival (PFS) in glioblastoma patients (39). In addition to improving migratory capacity, researchers have also targeted apoptotic pathways by promoting Bcl-2 or inhibiting BAK/BAX signaling in DCs to increase the lifetime of DCs and thereby enhance bioavailability of the injected DCs, which resulted in improved activation of T-cells (40–43). However, despite these attempts to improve DC-therapy, combinatorial strategies are essential to prorogue suppressive mechanisms in the TME and to further potentiate the clinical efficacy of DC-therapy.

## Combination Therapies to Enhance DC-Therapy Efficacy

### Combination With Chemotherapy

Chemotherapeutics are traditionally designed to eradicate and eliminate malignant cells to lower tumor burden. However, more recent insights indicate that chemotherapy also has off-target immunological effects depending on the type of chemotherapy, such as immunogenic cell death (ICD) of tumor cells, thereby enabling the induction of anti-tumor immunity (44). ICD stimulates emission of DAMPs, including adenosine triphosphate (ATP), high mobility group box 1 (HMGB1), and calrecticulin (CALR), which initiates antigen uptake, maturation, activation, and recruitment of endogenous DCs in the tumor (45, 46). In addition, specific chemotherapeutics can directly deplete suppressive immune cells including Tregs and MDSCs (47–49). Due to the effects on tumor burden and the immunosuppressive TME, chemotherapeutics could have synergistic effects when combined with DC-therapy. For instance, tumor reduction by neo-adjuvant chemotherapy could improve DC-therapy, as DC-therapy is most effective in cases of low-tumor burden (31). In addition, depletion of immunosuppressive cells in the TME renders the TME more receptive for tumor-specific T-cell infiltration upon DC-therapy. Timing of chemotherapy administration may be crucial as potential synergistic effects of combination treatments depend on the interval and sequence of treatment administration (50). For instance, chemotherapy applied prior to DC-therapy with substantial intervals aims at tumor reduction whereas shorter intervals or concurrent combination therapy allow depletion of suppressive immune cells. In the following sections, combinations of well-studied chemotherapeutics with *ex vivo* antigen-loaded DCs will be discussed. A summary of the main characteristics of the studies is presented in Table 1.

**Table 1:** Study characteristics of (pre)clinical studies.

**Type of CTX**	**Cancer type**	**n^a^**	**Comparison group**	**Treatment schedule**	**Type of DC vaccine**	**Dosage CTX**	**Immunological response CTX^c^**	**Immunological response combination treatment^c^, ^d^**	**Clinical response**		**References**
**PRE-CLINICAL**											
Cyclophosphamide	Mesothelioma (AB1)	6	Untreated CTX + DC-Tx + CTX DC-Tx + CTX	CTX: day 3–10^b^ DC-Tx: day 12^b^	Tumor lysate-loaded mature BM-derived DCs	0,13 mg/ml (drinking water)	↓ Tregs		Prolonged survival compared to untreated		(51)
	Melanoma (B16)	10	Untreated CTX DC-Tx	CTX: day 5^b^ DC-Tx: day 9 and 23^b^	Tumor lysate-loaded mature BM-derived DCs	50 mg/kg body weight			Prolonged survival compared to monotherapy and untreated		(52)
	Colon carcinoma (CT26)	10	Untreated CTX DC-Tx	CTX: day 5^b^ DC-Tx: day 9 and 23^b^	Tumor lysate-loaded mature BM-derived DCs	50 mg/kg body weight	↓ Tregs	↑ IFN-γ secreting lymphocytes	Prolonged survival compared to monotherapy and untreated		(52)
Gemcitabine	Pancreatic cancer (Panc02)	6–8	Untreated CTX DC-Tx	CTX 2 days prior and after DC-Tx for 5 weeks	BM-derived mature DCs loaded with Panc02 cells	25 and 50 mg/kg body weight			Prolonged survival compared to untreated (for both dosages)		(53)
	Pancreatic cancer (Panc02)	8	Untreated CTX DC-Tx	CTX: every 3-4 days until day 42 (start day 3) DC-Tx: day 3, 7 and 10^b^	Unloaded immature BM-derived DCs	120 mg/kg body weight	↓ MDSCs	↑ IFN-γ secreting lymphocytes ↑ CD8+ T-cells in tumor tissue	Prolonged survival compared to monotherapy and untreated		(54)
**CLINICAL**											
Cyclophosphamide	Melanoma	7		CTX: 3 days prior to first DC-tx. DC-tx: 6 vaccinations with 3-week intervals	gp100 antigen derived peptide-loaded mature autologous DCs	300 mg/m^2^		•T-cell immunity against gp100-derived antigens 6/7 •Positive correlation DC derived IL-12p70 levels and time to progression			(55)
	Mesothelioma	10		7x CTX followed by 1x DC-Tx 4 days after CTX. Cycle repeated 3x	Tumor lysate-loaded mature autologous DCs	2 × 50 mg	↓ Tregs		Disease control in 8 patients		(56)
	Melanoma	22		7x CTX followed by 1x DC-Tx. Cycle repeated 6x	Mature autologous DCs transfected with p53, survivin and hTERT	50 mg		•Tregs and MDSCs unchanged •IFN-γ Immune response 6/17	PD: *n =* 13 SD: *n =* 9	OS: 10.4 mo PFS: 3.1 mo	(57)
	Ovarian cancer	22	DC-tx(+ bevacizumab) (*n =* 10)	CTX one day prior to each DC-Tx + bevacuzimab given 1x each 3 weeks Repeated 4-5x	Tumor-lysate loaded mature autologous DCs	200 mg/m^2^		↑ Vaccine-specific T-cells ↑ IFN-γ serum levels ↓ TGF-β serum levels compared to no CTX	Improved OS compared to no treatment with CTX		(58)
	Renal cell carcinoma	22	DC-tx(*n =* 12)	CTX: 3 and 4 days prior to each DC-Tx DC-Tx: 3 vaccinations with monthly intervals	Tumor lysate-loaded mature allogeneic DCs	300 mg/m^2^		•No proliferative or cytokine immune responses	No CTX	CTX	(59)
									PD: *n =* 9	PD: *n =* 4	
									SD: *n =* 2	SD: *n =* 1	
									MR: *n =* 0	MR: *n =* 2	
									LFU: *n =* 1	LFU: *n =* 3	
									OS: 20.3 mo	OS: 23.2 mo	
Temozolomide	Melanoma	21		14x CTX followed by 1x DC-tx. Cycle repeated 6x	Tumor lysate-loaded mature autologous DCs	75 mg/m^2^		↓ Tregs	PD: *n =* 10	OS: 10 mo	(60)
									SD: *n =* 6		
									PR: *n =* 1		
									NT: *n =* 3		
	Glioblastoma	32		CTX: 5 days/28 in each cycle DC-Tx: 3x starting 2 weeks after CTX. Repeated 3x	DCs fused with glioma cells	150–200 mg/m^2^		•WT-1, gp100 and MAGE-A3 specific immune responses 4/4	Recurrent	Initial	(61)
									OS: 18.0 mo	OS: 30.5 mo	
									PFS: 10.3 mo	PFS: 18.3 mo	
	Glioblastoma	14		CTX: 5 days/28 starting one week after 3rd DC-Tx Cycle repeated up to 6x DC-Tx: 3x each cycle with 2 weeks intervals.	Tumor cell-loaded mature autologous DCs	150–200 mg/m^2^			PD: *n =* 4SD then PD: *n =* 3PR then PD: *n =* 2NT: *n =* 4	OS: 23 moPFS_6mo_: 22%	(62)
	Glioblastoma	24		CTX: 5 days/28 starting after 3rd DC-Tx. Cycle repeated 6x DC-Tx: 1-4: 2-weeks intervals, 5-6: monthly intervals, 7: 8 weeks after 6th DC-Tx	Tumor lysate-loaded mature autologous DCs	75 mg/m^2^		•Positive correlation activation NK cells and PFS	OS: 20.1 moPFS_:_ 10.5 mo		(63)
Gemcitabine	Pancreatic cancer	10		CTX: day 1,8 and 15 of a 28-days cycle DC-Tx: Starting one week after first CTX cycle. Given 3x biweekly	I, II or I/II-WT1 restricted peptide-loaded mature DCs	1,000 mg/m^2^			PD: *n =* 3SD: *n =* 7		(64)
Premetrexed and cisplatin	Mesothelioma	10		CTX: 4x each 3 weeks DC-Tx: 3x each 2 weeks starting 12 weeks after last CTX	Tumor lysate-loaded mature autologous DCs	Premetrexed: 500 mg/m^2^ Cisplatin: 75 mg/m^2^		↑ KLH-specific antibodies 10/10	PD: *n =* 6SD: *n =* 1PR: *n =* 3		(65)
Oxiplatin and capecitabine	Colon cancer	7		CTX: 1x oxiplatin followed by 14x capecitabine. Cycle repeated 8 times DC-Tx: 3x during first cycle of CTX	CEA peptide-loaded mature autologous DCs	Oxiplatin: 130 mg/m^2^ Capecitabine: 2,000 mg/m^2^		•CEA-specific T-cell response 4/7 •Proliferative KLH-specific CD4^+^ T-cell response 7/7			(66)
Bortezomib and dexamethasone	Multiple myeloma	50	CTX (*n =* 24)	Bortezomib: day 1,4,8, and 11 Dexamethasone: day 1-2, 4-5, 8-9, 11-12 DC-Tx: 6x day 15-20 Cycle lasted 28 days. Repeated 3x	Autologous DCs/CIK	Bortezomib: 1.0-1.3 mg/m^2^ Dexamethasone: 20 mg		↑ CD4/CD8 ratio ↑IL-2 and IFN-γ in PB ↓ IL-4, IL-5 and TGF-β in PB compared to CTX	Improved quality of life compared to no DC-Tx		(67)
Dacarbazine	Melanoma	6		CTX: 6x at 3-week intervals DC-Tx: 6x one day after CTX	Autologous IFN-DCs	1,000 mg/m^2^		•Tyrosinase, NY-ESO-1 and gp100-specific immune response 2/3	PD: *n =* 2SD: *n =* 3NT: *n =* 1		(68)
Carboplatin and paclitaxel	Melanoma	9		CTX: day 1 of each cycle DC-Tx: day 8 and 22 of each cycle Cycle lasted 28 days Repeated 3x	WT1, gp100, tyrosinase, and MAGE-A2/A3 peptide-loaded mature DCs	Carboplatin: AUC5 Paclitaxel: 175 mg/m^2^		•WT1-specific immune response 4/9	PD: *n =* 4SD: *n =* 4PR: *n =* 1	OS: 12 moPFS: 2.3 mo	(69)
Docetaxel	Prostate cancer	40	CTX(*n =* 19)	CTX: 1x each 3 weeks. Repeated 10x DC-Tx: 2x in cycle 1-5 and 1x cycle 5-10	Mature autologous DCs transfected with PSA, PAP, survivin and hTERT	75 mg/m^2^	•MDSCs andTregsunchanged	↓ MDSCs (positive correlation with PFS) •Tregs unchanged	PFS without DC-Tx: 5.5 moPFS with DC-Tx: 5.7 mo		(70)
	Esophageal cancer	10		CTX: day 1 of each cycle DC-Tx: day 15 and 22 of each cycle Cycle lasted 4 weeks. Repeated 3x	WT-1 peptide-loaded matured DCs	50 mg/m^2^		•WT1-specific immune response 5/8	PD: *n =* 9SD: *n =* 1OS: 5 mo		(71)

*AUC, area under curve; BM-derived DCs, bone marrow-derived DCs; CEA, carcinoembryonic antigen; CTX, chemotherapy; CIK, cytokine-induced killer cells; DC, dendritic cell; DC-Tx, dendritic cell therapy; gp100, glycoprotein 100; hTERT, human telomerase reverse transcriptase; IFN, interferon; IFN-γ, interferon gamma; IL, interleukin; KLH, keyhole limpet hemocyanin; LFU, lost to follow-up; MAGE, melanoma-associated antigen; MDSC, myeloid-derived suppressor cell; MR, mixed response; NK cells, natural killer cells; NT, not treated; OS, overall survival; PAP, prostatic acid phosphatase; PB, peripheral blood; PD, progressive disease; PFS, progression-free survival; PR, partial response; PSA, prostate-specific antigen; SD, stable disease; TGF-β, transforming growth factor beta; Tregs, regulatory T-cells; WT, wilms tumor gene*.

a *For preclinical studies n is number mice/group, for clinical studies n is the total number patients*.

b *Days after tumor inoculation*.

c *Compared to baseline unless indicated otherwise*.

d *immunological responses measured after combination treatment*.

#### Cyclophosphamide

**Figure 1:**
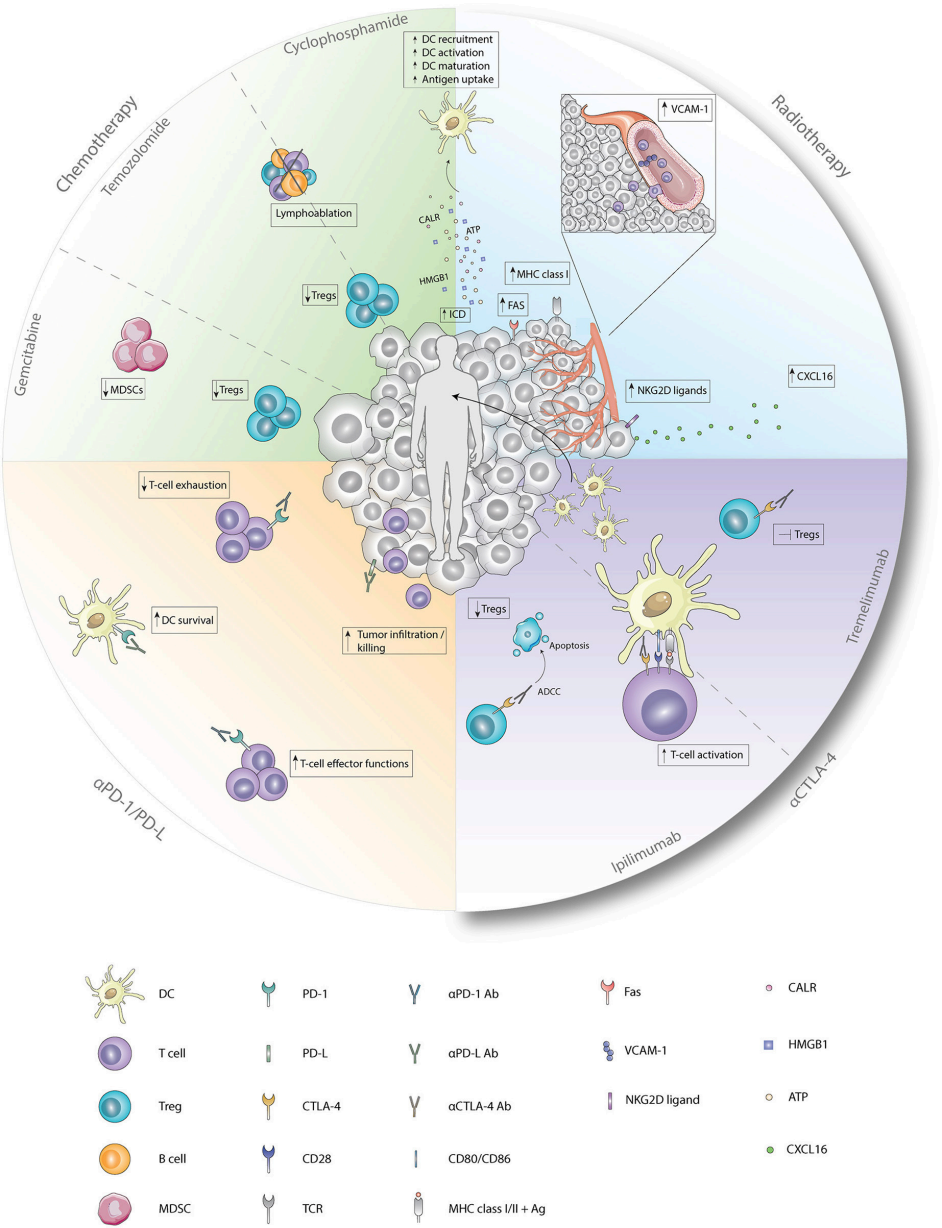
Immunological effects of chemotherapy, radiotherapy, and checkpoint inhibitors. Cyclophosphamide induces ICD which enhances the recruitment, activation, maturation, and antigen uptake by DCs. In addition, cyclophosphamide and temozolomide deplete Tregs and induce lymphoablation upon treatment with low-dose or high-dose, respectively. Immunological functions of gemcitabine entail depletion of Tregs and MDSCs. Radiotherapy induces, besides ICD, enhanced expression of FAS, MHC class I, and NKG2D ligands on tumor cells and enhanced expression of VCAM-1 on endothelial cells. Furthermore, secretion of CXCL16 by tumor cells is increased after radiotherapy. Antagonistic CTLA-4 antibodies enhance T-cell activation by the preventing the binding of CD28 with CD80/86. Ipilimumab depletes Tregs by ADCC whereas tremelimumab inhibits functions of Tregs upon binding. Anti-PD1 antagonistic antibodies enhance T-cell effector functions while preventing exhaustion of T-cells. Blockade of PD-1 on DCs improves survival while blockade of PD-L on tumor cells results in improved tumor-cell infiltration and killing. Ab, antibody; Ag, antigen; ATP, adenosine triphosphate; CALR, calreticulin; CTLA-4, cytotoxic T-lymphocyte-associated antigen; CXCL16, chemokine ligand 16; DC, dendritic cell; Fas, first apoptosis signal; HMGB1, high mobility group box 1; MDSC, myeloid-derived suppressor cell; MHC class I/II, major histocompatibility complex class I/II; NKG2D ligand, natural killer group 2 member D; PD-1, programmed death 1; PD-L, programmed death ligand; TCR, T-cell receptor; Treg, regulatory T cell; VCAM-1, vascular endothelial cell adhesion protein 1.

Cyclophosphamide is an alkylating agent that has tumoricidal effects, thereby reducing tumor burden (72). In addition, cyclophosphamide initiates ICD and transient lymphoablation upon high doses, thereby resulting in depletion of suppressive immune cells and stimulation of anti-tumor T-cell responses. In contrast, low-dose cyclophosphamide improves tumor-specific immunity by Treg depletion (Figure 1) (47). In mesothelioma, melanoma and colon carcinoma murine models, administration of cyclophosphamide prior to DC-therapy prolonged survival compared to mice treated with monotherapy. This is likely caused by a cyclophosphamide-induced decrease in Tregs, and subsequent increase in T-cells, as observed in these studies (51, 52). Cyclophosphamide administration 3 days prior to DC-therapy was shown to induce T-cell responses to 3 melanoma gp100 antigen-derived peptides G154, G206-2M, and G280-GV in 6 out of 7 melanoma patients post vaccination (55). A reduction in Tregs was also observed in mesothelioma patients treated with concurrent combination of cyclophosphamide and DC-therapy but remained unaffected in a study with melanoma patients (56, 57). These differences could be explained by differences in sampling time, as reduction in Tregs was evaluated after the first cyclophosphamide treatment in mesothelioma patients (56), whereas in melanoma patients, these levels were assessed after 4 and 6 cycles of DC-therapy (57). Combining DC-therapy with cyclophosphamide also improves clinical efficacy, as patients with ovarian cancer that received cyclophosphamide concurrent with DC-therapy and bevacizumab, a VEGF-a blocking antibody, exhibited significantly prolonged survival compared to patients without cyclophosphamide treatment (58). These results were associated with reduced TGF-β levels, a cytokine that is abundantly produced by Tregs in ovarian cancer. Contradictory, combined DC-therapy with cyclophosphamide resulted in poor clinical responses in patients with metastatic renal cell carcinoma. However, as the DCs administered in this study were of allogeneic origin, the lack of clinical efficacy could be explained by the nature of the DCs administered (59). These results indicate that Treg depletion upon cyclophosphamide treatment is able to synergistically augment DC-therapy efficacy both in preclinical and clinical settings, depending on the tumor type and DCs applied.

#### Temozolomide

The alkylating agent temozolomide (TMZ) induces lymphoablation upon high doses whereas at low doses it primarily targets Tregs (Figure 1) (49). As this compound effectively crosses the blood-brain barrier, TMZ is mainly used to treat glioblastoma and melanoma, as the brain is a frequent metastatic site for melanoma (73, 74). In patients with advanced melanoma, administration of one TMZ cycle prior to each DC-therapy decreased circulating Tregs with 60.5% (60). Simultaneous administration of TMZ and DC/glioma cell fusions in recurrent and newly-diagnosed glioblastoma patients resulted in WT-1, gp100, and MAGE-A3-specific CTLs upon vaccination. In the newly-diagnosed patients, PFS and OS were improved compared to an international trial of TMZ monotherapy (61). However, in recurrent glioblastoma patients, where DC-therapy was followed by TMZ administration, combined treatment failed to improve 6-month PFS compared to a reference group with TMZ monotherapy (62). This could be due to reduced CTL numbers caused by TMZ-induced lymphoablation, thereby counteracting the effects of DC-therapy, as shown by a recent study (63). Interestingly, this study also illustrated that, in contrast to CTL numbers, NK cells in peripheral blood remained constant after concurrent combinations with TMZ. However, whether the effects observed on NK cells were associated with depletion of Tregs remains elusive. Furthermore, this indicates that TMZ administration before or during DC-therapy could enhance DC-therapy efficacy, whereas DC-therapy followed by TMZ may exert negative effects on DC-induced anti-tumor immunity.

#### Gemcitabine

Gemcitabine is able to improve anti-tumor immunity by depletion of MDSCs and Tregs (Figure 1) (47, 48, 75). Treatment of mice bearing pancreatic tumors with gemcitabine 2 days before and after DC-therapy prolonged survival compared to untreated mice, which was not observed for both monotherapies (53). Concurrent treatment of DC-therapy and gemcitabine in a murine pancreatic model delayed tumor growth and prolonged survival compared to both monotherapies. This could be dependent on MDSC numbers, as MDSC numbers were significantly reduced in spleens and tumors of mice treated with gemcitabine (54). However, in pancreatic cancer patients, despite decreased PD-1^+^CTL numbers in responders, the concurrent combination did not result in decreased MDSC and Treg numbers in responders vs. non-responders (64). These results indicate that gemcitabine may enhance DC-therapy efficacy, however the mechanism of action warrants further investigation.

#### Combination With Other Chemotherapies

With the aim to reduce tumor burden, Hegmans et al. treated mesothelioma patients with premetrexed and cisplatin 12 weeks prior to DC-therapy, which resulted in immunological responses in all patients against keyhole limpet hemocyanin (KLH), a protein used to assess T-cell responses initiated by DC-therapy (65). As this trial has no control arm no conclusions on synergy can be made. Co-administration of oxiplatin, capecitabine and DC-therapy in colon cancer patients induced proliferation of KLH-specific CD4^+^ T-cells in all patients as well (66). An effect on CD4^+^ T-cells was also observed in multiple myeloma patients wherein treatment with DCs and cytokine-induced killer cells (CIK) combined with bortezomib and dexamethasone improved CD4^+^/CD8^+^ T-cell ratios compared to baseline and treatment with chemotherapy alone (67). Specific anti-tumor immunity with CTLs directed against gp100, tyrosine and NY-ESO was induced in 67% of the patients with advanced melanoma treated with the combination of DC-therapy and dacarbazine (68). In addition, in 44% of the patients with stage IV melanoma, a specific immune response against WT1 was induced upon treatment with DC-therapy and carboplatin and paclitaxel (69). However, combination with docetaxel failed to improve clinical responses in patients with esophageal cancer and did not result in improved PFS in patients with prostate cancer compared to docetaxel monotherapy (70, 71). These results indicate that combined treatment with chemotherapy and DC-therapy is feasible and safe, however further research should be conducted providing insight into the potential synergistical effects.

### Combination With Radiotherapy

Ever since radiotherapy was found to affect non-radiated tumor lesions in a process called the abscopal effect, the immunomodulatory effects of this therapy have been more thoroughly appreciated. As radiotherapy induces ICD, one primary effect is the release of DAMPs and tumor-derived antigens, thereby initiating the activation and migration of DCs to the LN where DCs subsequently cross-present these antigens to T-cells and induce systemic anti-tumor immune responses (Figure 1) (76–80). The induction of systemic anti-tumor immunity was indeed observed when radiotherapy was combined with GM-CSF as it generated abscopal effects in some patients (81). In addition, the combination with Flt-3 ligand in a Lewis lung carcinoma murine model reduced metastases and prolonged survival (82). However, in settings of compromised DC functionality, intratumoral injection of exogenously-prepared unloaded DCs followed by radiotherapy could be advantageous. Induction of systemic immunity was observed in a squamous-cell carcinoma murine model, as combining radiotherapy with intratumoral DC administration increased the presence of CTLs in the tumor-draining LN (TDLN) compared to DC-monotherapy (83). In addition, reduced tumor burden and prolonged survival were observed compared to monotherapy in multiple preclinical models (84–88). In clinical trials with patients suffering from hepatocellular carcinoma and high-risk sarcoma, combining intratumoral injection of unloaded DCs with radiotherapy induced tumor-specific immunity in 70 and 52.9% of the cases, respectively (89, 90). In addition to induction of synergistic effects when combined with unloaded DCs, radiotherapy may also improve efficacy when combined with loaded DCs as it transforms irradiated tissue into an immunogenic niche by enhancing the expression of vascular endothelial cell adhesion protein 1 (VCAM-1) on endothelial cells, FAS, MHCI and natural killer group 2D (NKG2D) on tumor cells and increasing CXCL16 secretion, thereby promoting homing, infiltration and tumor killing by DC-induced lymphocytes (Figure 1) (91–96). In patients with stage I esophageal cancer, 1- and 2-year survival were significantly improved upon treatment with loaded DCs and radiotherapy as compared to radiotherapy alone. Addition of CIK to this combination failed to improve survival in patients with stage III/IV non-small-cell lung cancer (97, 98). These results indicate that combinatorial treatment has synergistic effects, but these depend on tumor type and stage, as improved efficacy is most prominent at early tumor stages.

### Combination With Immune Checkpoint Inhibitors

In cancer, tumor cells and immune cells often overexpress co-inhibitory molecules, such as PD-1/PD-L1 and CTLA-4, which suppress anti-tumor immunity. Checkpoint inhibitors targeting these co-inhibitory molecules improve existing anti-tumor immunity when administered as monotherapy (99, 100). Additionally, combinations with DC-therapy may result in synergistic effects as expression of these co-inhibitory molecules could also limit durable DC-therapy effects by inhibiting DC-therapy induced T-cells as well as DCs directly.

#### PD-1/PD-L Blocking Antibodies

The PD-1/PD-L-axis exerts negative effects on TME-infiltrating immune cells by inhibiting T-cell effector functions, NK cells and inducing T-cell exhaustion (101–104). Additionally, PD-L1 expression on tumor cells also directly inhibits IFN-γ-mediated cytotoxicity by a STAT3/caspase 7 dependent pathway (105). Therapeutically targeting PD-1/PD-L1 could therefore render the TME more receptive for lymphocyte infiltration and sensitize tumor cells for cytotoxicity that could act synergistically upon combination with DC-therapy (Figure 1). Combining DC-therapy with PD-1 blockade reduced Tregs, induced IFN-γ secretion, while secretion of IL-10 by CD4^+^ T-cells was decreased. In addition, cytotoxicity of CTLs improved when PD-1 was inhibited in a co-culture of tumor cells and T-cells isolated from mice treated with DC/myeloma fusions (106). *In vivo* investigation of DC-therapy combined with PD-1 blockade reduced tumor volume of mice with melanoma (107) and prolonged survival in murine models for glioblastoma (108) compared to monotherapy. These beneficial effects on anti-tumor immunity were also observed in a breast cancer murine model upon combinations with anti-PD-L1 antibodies (109). Additionally, this study investigated the combination of specific blockade of PD-L1 on DCs by *in vitro* incubation with antagonistic monoclonal antibodies (109).

PD-L1/2 are both expressed on DCs and are associated with suppression of effector CTLs and CD4^+^ T-cells and induction of Treg expansion (110–117). Conversely, the expression of PD-1 on DCs negatively affects DC survival (118). This indicates that blockade of PD-1 or PD-L1 on DCs could enhance anti-tumor immunity *in vivo* via multiple ways. PD-L1 blockade on DCs improved maturation and proliferation of DCs during culture, inhibited tumor outgrowth and prolonged survival compared to mice treated with DCs on which PD-L1 was not blocked (109). These results underline the importance of PD-L1 expression on DCs in inhibiting anti-tumor immunity. Therefore, efforts are undertaken to establish DC-specific PD-L1 blockade, primarily by different RNA introducing techniques, such as small interference RNA (siRNA) or short hairpin RNA (shRNA). Preclinical data indicate that PD-L1 can effectively be silenced using these approaches without affecting viability, maturation or costimulatory molecule expression. In addition, silencing PD-L1 or PD-L2 specifically on DCs enhanced proliferation of tumor-specific CTLs and CD4^+^ T-cells, augmented production of IFN-γ, tumor-necrosis factor alpha (TNFα), IL-2, IL-5, and IL-12 and promoted cytolysis of tumor cells *in vitro* (119–123). These promising data provide incentive to further investigate the combination of systemic PD-(L)1 blockade with DC-therapy and PD-L1 blockade on DCs.

#### CTLA-4

The antagonistic antibodies ipilimumab and tremelimumab are designed to target CTLA-4, an inhibitory pathway that inhibits activation of naïve T-cells by preventing the binding of CD28 on T-cells to CD80/CD86 on APCs, a mechanism widely exploited by Tregs (124, 125). In various murine models, ipilimumab was shown to induce antibody-dependent cell-mediated cytotoxicity (ADCC), thereby facilitating Treg depletion while tremelimumab inhibits effector functions of Tregs (Figure 1) (126, 127). However, recent clinical data question the Treg-depleting capacity of ipilimumab, as treatment with ipilimumab did not deplete Tregs in the TME of patients with melanoma, prostate cancer and bladder cancer (128). In a retrospective study with stage III melanoma patients that progressed after DC-therapy, administration of ipilimumab induced tumor-specific T-cell responses in 72% of the cases although this was not associated with improved OS (129). Clinical and CTL responses were also not associated in a clinical trial with 16 melanoma patients treated with MART-1 peptide loaded DCs and tremelimumab (130). However, most promising clinical results were obtained by a recent study, in which the overall response rate reached 38% in advanced melanoma patients. These patients were treated with ipilimumab combined with DCs electroporated with CD40L, CD70, and constitutively activated TLR-4 encoding mRNA and one of 4 melanoma-associated antigens (MAGE-A3, MAGE-C2, tyrosinase, or gp100) fused to an HLA-class II targeting signal (131). This indicates that combining DC-therapy with CTLA-4 targeting agents could lead to synergistic effects.

### Combination With Other Immunomodulating Therapies

Recently, also other immunomodulatory therapies were approved that enable depletion of specific immunosuppressive cell types, such as macrophages that are depleted upon antibody or tyrosine kinase inhibition of the M-CSF-receptor. In line, we have previously combined DC-therapy with M-CSFR inhibitor treatment in murine tumor models and found improved survival compared to DC-monotherapy. In addition, numbers, proliferation and exhaustion state of CTLs were improved (132). Similar results were obtained when combining DC-therapy with a CD40-agonistic antibody, capable of converting macrophages to a proinflammatory phenotype, and further stimulating the CD40^+^DCs (133). Besides macrophages, selective depletion of Tregs could enhance anti-tumor immunity. Results in a preclinical melanoma mouse model showed that depletion of Tregs using anti-CD25 antibodies prior to DC-therapy elicits long-lasting anti-tumor immunity, as most mice remained tumor-free after tumor rechallenge (134). Further investigation into these combinations in different (pre)clinical models could lead to promising novel combination strategies.

## Future Perspectives

Despite the clinical success of DC-therapy, clinical efficacy remains limited to a proportion of patients and integration of combinatorial approaches are therefore warranted to improve efficacy. Timing of these combinatorial approaches should be carefully considered as this will affect the potential synergistic mode of action. In addition, determining optimal combination therapies likely depends on multiple factors including patient's condition, tumor type, stage and composition of the TME. Therefore, characterization of tumor cells and immune cells present in the TME or peripheral blood of individual patients will help to select immunotherapies that most likely will work synergistically with DC-therapy. For example, treatment of tumors enriched with Tregs should entail combinations with Treg-depleting chemotherapeutics, whereas DC-therapy should be combined with PD-L1 antagonistic antibodies in tumors with high PD-L1 expression. Furthermore, careful characterization of the TME, and peripheral blood could provide novel insights for combination strategies.

## Conclusion

Although combinations with DC-therapy have demonstrated beneficial effects contributing to anti-tumor immunity, the potential for further improvement remains. A major focus should be on the careful characterization of tumor and peripheral blood of each individual patient as this will be needed to tailor treatments and enhance efficacy on a personalized level. In addition, more controlled clinical trials should be executed to directly compare efficacy with monotherapy. Timing of treatment administration should be taken into consideration in these studies as it could affect the efficacy of combination therapies.

## Author Contributions

MvG and HV wrote the manuscript and generated the figure and table. FD and JA contributed to the revisions of the manuscript. All authors approved the manuscript for publication.

### Conflict of Interest Statement

JA: No relationship to disclose in relation to the submitted work. Relevant financial activities outside the submitted work: Stock or Other Ownership: Amphera Consulting or Advisory Role: Bristol-Myers Squibb, MSD Oncology, Boehringer Ingelheim, Eli-Lilly, Roche Speakers Bureau: AstraZeneca. Research Funding: Genentech (Inst), Boehirnger Ingelheim (inst). Patents, Royalties, Other Intellectual Property: Patent: Tumor cell lysate for dendritic cell loading (Inst), SNP analyses for immunotherapy (Inst). The remaining authors declare that the research was conducted in the absence of any commercial or financial relationships that could be construed as a potential conflict of interest.
